# An Autism Spectrum Disorder Identification Method Based on 3D-CNN and Segmented Temporal Decision Network

**DOI:** 10.3390/brainsci15060569

**Published:** 2025-05-25

**Authors:** Zhiling Liu, Ye Chen, Xinrui Dong, Jing Liu

**Affiliations:** 1 Faculty of Psychology, Beijing Normal University, Beijing 100875, China; zhiling.liu@mail.bnu.edu.cn; 2 College of Computer Science, Beijing University of Technology, Beijing 100124, China; chenye74008@emails.bjut.edu.cn (Y.C.); xinruidong@emails.bjut.edu.cn (X.D.)

**Keywords:** autism spectrum disorder, machine learning, FMRI, ABIDE

## Abstract

(1) Background: Autism Spectrum Disorder (ASD) is a neurodevelopmental disorder characterized by social communication deficits and repetitive behaviors. Functional MRI (fMRI) has been widely applied to investigate brain functional abnormalities associated with ASD, yet challenges remain due to complex data characteristics and limited spatiotemporal information capture. This study aims to improve the ability to capture spatiotemporal dynamics of brain activity by proposing an advanced framework. (2) Methods: This study proposes an ASD recognition method that combines 3D Convolutional Neural Networks (3D-CNNs) and segmented temporal decision networks. The method first uses the 3D-CNN to automatically extract high-dimensional spatial features directly from the raw 4D fMRI data. It then captures temporal dynamic properties through a designed segmented Long Short-Term Memory (LSTM) network. The concatenated spatiotemporal features are classified using Gradient Boosting Decision Trees (GBDTs), and finally, a voting mechanism is applied to determine whether the subject belongs to the ASD group based on the prediction results. This approach not only enhances the efficiency of spatiotemporal feature extraction but also improves the model’s ability to learn complex brain activity patterns. (3) Results: The proposed method was evaluated on the ABIDE dataset, which includes 1035 subjects from 17 different brain imaging centers. The experimental results demonstrate that our method outperforms existing state-of-the-art approaches, achieving an average accuracy of 0.85. (4) Conclusions: Our method provides a new solution for ASD classification by leveraging the spatiotemporal information of 4D fMRI data, achieving a significant improvement in classification performance. These results not only offer a new computational tool for ASD diagnosis but also provide important insights into understanding its neurobiological mechanisms.

## 1. Introduction

Autism Spectrum Disorder (ASD) is a neurodevelopmental disorder characterized by core features of social communication deficits and repetitive, restrictive behaviors [1]. Epidemiological studies indicate that approximately 1 in 100 children worldwide are diagnosed with autism, leading to a significant burden on both families and society [2]. Currently, the clinical diagnosis of ASD primarily relies on the criteria outlined in the Diagnostic and Statistical Manual of Mental Disorders (DSM-5) and is supplemented by behavioral assessment tools such as the Autism Diagnostic Observation Schedule (ADOS) [3]. However, this diagnostic approach is subject to inherent limitations due to its subjective nature and is unable to uncover the underlying neurobiological mechanisms of the disorder.

The application of magnetic resonance imaging (MRI) in ASD research has been gradually increasing, particularly in the exploration of brain structural and functional abnormalities. These studies have provided valuable insights into the neurodevelopmental characteristics of ASD. However, despite the fact that MRI techniques have revealed brain structural and functional changes associated with ASD, these findings have often not been consistently validated across all ASD patients [4]. Structural MRI (sMRI) is primarily used to observe static anatomical features of the brain, focusing on metrics such as brain region volumes, morphology, and the density of gray and white matter in order to identify potential structural abnormalities. However, such studies tend to focus on morphological changes and are often unable to fully capture the dynamic functional processes of the brain. In this regard, functional MRI (fMRI) has emerged as an important tool for investigating brain activity and neural network connectivity, offering a new avenue for exploring the neurobiological underpinnings of ASD. Through fMRI, researchers can observe brain activity patterns during different tasks or resting states, revealing functional cooperation and connectivity changes between different brain regions. This dynamic approach provides a unique perspective for understanding the neurobiological mechanisms of ASD, particularly in uncovering abnormal connectivity within brain functional networks, which holds irreplaceable advantages.

In the analysis of sMRI, Ecker et al. [5] employed a multi-parametric classification approach to characterize the complex and subtle gray matter anatomical patterns associated with autism in adults. Jiao et al. [6] utilized regional cortical thickness extracted from surface-based morphology to perform ASD classification, achieving good classification performance. Kong et al. [7] proposed a deep neural network classifier based on stacked autoencoders, which effectively classified ASD by extracting features from structural MRI images and performing feature selection. Ji et al. [8] proposed the use of 3D Convolutional Neural Networks to train whole-brain images and obtain relevant feature information. However, the high-dimensional nature of sMRI data presents significant challenges for computational hardware, making direct input into networks difficult. To address this, researchers divided the complete 3D images into smaller patches before inputting them into the network, a method that not only fully utilized whole-brain features but also significantly improved training efficiency. Puranik et al. [9] adopted a dimensionality reduction strategy by slicing 3D sMRI images into 2D slices, combined with transfer learning techniques for classification, which effectively alleviated computational pressure. However, due to the heterogeneity of brain structures in ASD patients, the generalization ability of such methods remains limited.

In the analysis of functional MRI (fMRI), Nielsen et al. [10] achieved a classification accuracy of 60% based on whole-brain functional connectivity analysis, identifying connection patterns in key brain regions such as the default mode network as the most discriminative. While such methods are simple and effective, they have limitations, including reliance on manually designed feature extraction and the difficulty in capturing complex nonlinear relationships. With the development of deep learning techniques, researchers have begun to explore more complex model architectures. Heinsfeld et al. [11] employed deep neural networks to analyze multi-center brain imaging data, achieving a 70% accuracy in identifying ASD patients through functional connectivity patterns. Eslami et al. [12] utilized a joint learning framework combining autoencoders and single-layer perceptrons (SLPs) to optimize feature extraction and classification parameters, enhancing the performance of autism diagnostic models by incorporating a data augmentation strategy using linear interpolation in the feature space. Rakić et al. [13] combined the volume of regions of interest (ROIs) from structural MRI and functional connectivity matrices, training with autoencoders and multi-layer perceptrons, and achieved the highest classification accuracy of 93.18% at the CMU site. Although significant progress has been made in ASD classification based on single-site data, existing methods generally face the key challenge of limited generalization ability. To address this challenge, Epalle et al. [14] proposed an innovative multi-atlas feature fusion strategy. This study aligned the feature dimensions of resting-state fMRI data from three different sites using fully connected layers, constructing a unified multi-site feature representation space. The method achieved cross-site knowledge transfer through feature space alignment, offering a new approach to improving model generalization. Kang et al. [15] proposed a multi-center autism recognition method based on LeNet5 and MLP, achieving 93% accuracy in single-site classification and 83.5% accuracy in multi-center classification by using glass brain feature extraction and SMS data partitioning strategies.

Recently, several deep-learning-based methods have attempted to extract multi-dimensional features. Deng et al. [16] proposed the ST-Transformer, which incorporates a spatial–temporal multi-head attention mechanism to jointly capture spatial structures and temporal dynamics in fMRI signals while also addressing data imbalance through a Gaussian GAN-based augmentation strategy. Liu et al. [17] introduced a pseudo 4D ResNet model that decomposes spatiotemporal convolutions into parallel 3D spatial and 1D temporal blocks, reducing computational complexity while preserving essential spatiotemporal patterns. Alharthi et al. [18] explored multi-slice generation from both sMRI and fMRI modalities, applying 3D-CNNs along with vision transformer models and leveraging transfer learning to enhance ASD diagnosis under limited data conditions. Although these studies have made notable progress, they still face the following key issues:

Firstly, most studies reduce the dimensionality of 4D fMRI data or use only static functional connectivity matrices, thereby losing valuable spatiotemporal dynamic information. As a neurodevelopmental disorder, ASD-related brain dysfunction is often characterized by dynamic changes, and static analysis methods are insufficient to capture these subtle variations. Although certain studies have incorporated temporal features into their models, many still depend on manually crafted slice selection, downsampling procedures, or modality-specific adjustments. Such design choices, while enabling the extraction of spatial or temporal representations, may hinder the scalability of the methods and restrict their ability to capture fine-grained temporal dynamics. Secondly, there is significant heterogeneity in data across different scanning centers, including differences in scanning parameters, subject populations, and preprocessing pipelines, which severely impacts the generalization ability of models. Furthermore, existing methods insufficiently model the dynamic evolution of functional networks and lack effective utilization of temporal information.

To address these challenges, this study proposes an ASD recognition method that combines 3D Convolutional Neural Networks (3D-CNNs) and segmented temporal decision networks. The method first utilizes 3D-CNNs to automatically extract high-dimensional spatial features from raw 4D fMRI data, avoiding the loss of valuable information that may occur with traditional dimensionality reduction techniques. Furthermore, to effectively capture the temporal dynamics of brain activity, we designed a segmented Long Short-Term Memory (LSTM) network architecture that divides the time series into physiologically meaningful segments to capture the temporal characteristics of brain activity. Then, Gradient Boosting Decision Trees (GBDTs) are used to classify the concatenated spatiotemporal features. Finally, a voting mechanism integrates the predictions from different time segments to classify the subject as either ASD or typically developing. This method not only enhances the efficiency of spatiotemporal feature extraction but also strengthens the model’s ability to learn complex dynamic brain activity patterns while ensuring feature integrity. Overall, the main contributions of our work are as follows:

- This study proposes a method that combines 3D-CNNs with segmented LSTM networks to automatically extract high-dimensional spatial features from raw 4D fMRI data and capture the temporal dynamics of brain activity through segmented LSTM. This approach effectively leverages the rich information in spatiotemporal data, enabling joint modeling of both spatial and temporal information, thereby enhancing the model’s ability to understand complex brain activity patterns.
- This study employs GBDTs for classification and designs a voting mechanism to integrate predictions from different time segments, leading to the final ASD diagnosis. This approach not only enhances the model’s learning capacity but also improves the accuracy and efficiency of ASD identification.
- Experimental results on the ABIDE dataset show that our method outperforms existing state-of-the-art approaches, achieving an average accuracy of 0.85. Furthermore, we employed t-SNE dimensionality reduction to illustrate the improved discriminative ability of the spatiotemporal fused features in distinguishing between ASD and typical control groups.

We hypothesize that the proposed deep learning framework, which jointly models spatial and temporal features from fMRI data, will achieve superior classification performance compared to conventional baseline approaches. Furthermore, we expect the model to obtain a robust average classification accuracy exceeding 80% across multiple imaging sites, thereby demonstrating its potential applicability in clinical diagnostic support for ASD.

## 2. Materials and Methods

### 2.1. ABIDE Dataset Description

This study utilizes the publicly available Autism Brain Imaging Data Exchange I (ABIDE I) dataset as the research subject [19]. The ABIDE initiative, launched by 17 international research institutions, aims to integrate multi-site structural and functional magnetic resonance imaging data to provide large-scale sample support for neuroimaging research related to ASD. The ABIDE I dataset includes a total of 1112 participants, comprising 539 individuals diagnosed with ASD and 573 typical control (TC) participants. The age range of the subjects spans from 7 to 64 years, covering childhood, adolescence, and adulthood.

Each participant in the dataset includes at least one resting-state functional magnetic resonance imaging (rs-fMRI) scan and one T1-weighted structural MRI (MPRAGE) scan. In addition, a wealth of phenotypic information (e.g., gender, age, diagnostic methods, etc.) is provided, totaling 73 variables, which are used for further analysis of individual differences. Table 1 and Table 2 presents the class membership information for each acquisition site. It is important to note that, due to the multi-site nature of the ABIDE dataset, there are variations in scanning parameters (e.g., repetition time, echo time, voxel size, whether the eyes were closed, etc.), diagnostic criteria (e.g., ADOS and ADI-R), and acquisition equipment across different sites. These differences present challenges for cross-site data analysis.

### 2.2. Data Preprocessing

In the data preprocessing phase, this study primarily utilized the Nilearn package in conjunction with other commonly used preprocessing tools to ensure data quality and consistency. In contrast to commonly used preprocessing pipelines, which involve complex and often automated workflows with numerous optional modules, we adopted a more streamlined and controllable preprocessing strategy using Nilearn. This approach retains the essential steps but avoids the overhead of redundant or black-box procedures. Nilearn offers efficient image loading, processing, and analysis capabilities, making it particularly suitable for handling large-scale fMRI datasets [20]. The rs-fMRI data for all participants were initially read and processed using Nilearn, followed by spatial alignment and normalization, where all fMRI images were registered to the standard MNI152 template space. Specifically, the relevant function was used for spatial registration, ensuring consistency across image data from different subjects.

Although Nilearn provides powerful tools for image processing, it does not offer built-in methods for slice timing correction and motion correction. Therefore, this study combined Nilearn with the FSL package to perform these critical preprocessing steps. Specifically, FSL’s mcflirt function was used for motion correction to remove noise due to motion artifacts, while slice timing correction was performed using FSL’s tool to correct for temporal offsets between slices acquired at different time points.

After spatial alignment and motion correction, intensity normalization was performed to eliminate inconsistencies in intensity caused by scanner differences and other technical factors. Subsequently, global signal regression (GSR) was applied to remove interference from global brain activity, thereby enhancing the signal-to-noise ratio (SNR) of local brain regions.

To further improve the signal quality and remove low-frequency drift and high-frequency noise, band-pass filtering was applied. The frequency range for the filtering was set between 0.01 Hz and 0.1 Hz, aiming to remove low-frequency drifts and high-frequency noise unrelated to brain functional activity.

Finally, to ensure data quality and consistency, the mean framewise displacement (FD) for each participant was computed to quantify the impact of motion artifacts. If the mean FD exceeded 0.2 for a given participant, their data were considered to have significant motion artifacts and were excluded from the analysis. While the choice of this threshold helps maintain data integrity, it may indeed impact the representativeness of the sample, particularly considering factors such as patient age, gender, and symptom severity. We acknowledge that these biases may limit the generalizability of the results; however, the impact of motion artifacts on model learning capabilities is more critical in model training. In future research, we will consider using a more lenient threshold to include a more diverse participant sample or employ supplementary analytical methods to validate the robustness of the results. Meanwhile, for the screened data, we utilized Nilearn’s functions to remove motion artifacts and other potential noise components to ensure the quality of the data used for analysis. After completing these preprocessing steps, the final dataset consisted of 974 valid participants, including 458 individuals with ASD and 516 TC.

### 2.3. Methods

#### 2.3.1. Overall Framework

**Figure 1:**
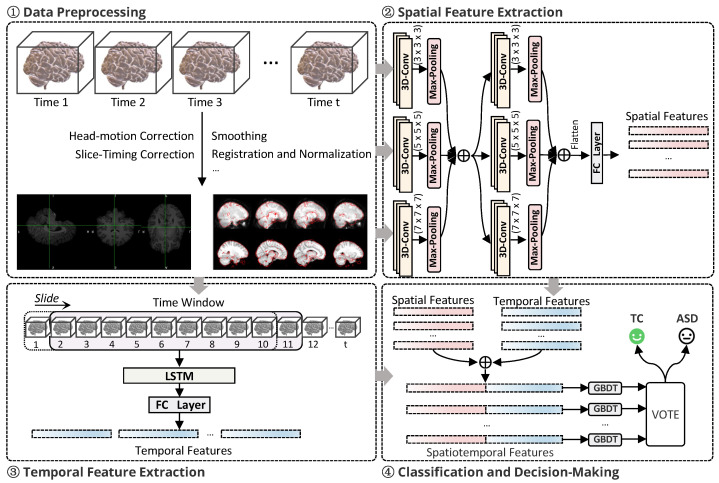
The overall framework of this study.

The overall framework of this study is illustrated in Figure 1, which consists of four main modules: (1) **Data Preprocessing**; (2) **Spatial Feature Extraction**; (3) **Temporal Feature Extraction**; (4) **Classification and Decision-Making**. These modules are closely integrated, forming a comprehensive pipeline for ASD recognition.

The data preprocessing module is considered a critical step to ensure the quality of input data. The raw 4D fMRI data were first loaded using the Nilearn toolkit, followed by spatial alignment and standardization. All fMRI images were registered to the standard MNI152 template space, ensuring spatial consistency across different subjects. Subsequently, slice timing correction and motion correction were performed using the FSL toolkit, removing motion artifacts. Intensity normalization and global signal regression were then applied to eliminate scanner-related differences and interference from global brain activity, thereby improving the signal-to-noise ratio (SNR) of local brain regions. Finally, a band-pass filter (0.01 Hz to 0.1 Hz) was applied to remove low-frequency drifts and high-frequency noise, ensuring that the data used for subsequent analysis possessed a high SNR.

In the spatial feature extraction module, a 3D-CNN was employed to automatically extract high-dimensional spatial features from the raw 4D fMRI data. The 3D-CNN directly processes the four-dimensional data, thereby avoiding potential information loss associated with traditional dimensionality reduction methods. The decision to utilize a design comprising two convolutional layers is based on a careful consideration of the trade-off between model complexity and performance. Preliminary experiments indicate that two convolutional layers are effective in capturing the functional connectivity patterns across different brain regions while maintaining a relatively low risk of overfitting, which also contributes to a more stable training process. Furthermore, the design of the 3D-CNN allows us to fully leverage spatiotemporal features, providing rich spatial information that lays the groundwork for subsequent temporal feature extraction.

In the temporal feature extraction module, a segmented LSTM network architecture was utilized to capture the dynamic temporal characteristics of brain activity. Compared to Gated Recurrent Units (GRUs), LSTM networks are more adept at capturing long-term dependencies when processing time-series data, particularly in the context of complex fMRI datasets. We selected 500 hidden units to control the model size and facilitate the training tasks on the available hardware. This dimensionality has been found sufficient for effectively capturing the dynamic features of brain activity. Additionally, in the classification phase, we employed the Gradient Boosting Decision Tree (GBDT) as our ensemble learning method, rather than opting for the more complex XGBoost. The GBDT demonstrates excellent performance in handling high-dimensional features, effectively addressing the issue of model overfitting while also offering good interpretability, allowing us to understand the basis of the model’s decisions. Overall, the combination of a two-layer 3D-CNN, segmented LSTM, and GBDT strikes a favorable balance between accuracy and model complexity, making it well suited for our application in ASD classification tasks.

In the classification decision module, the GBDT was employed to classify the concatenated spatiotemporal features. The GBDT integrates multiple weak classifiers to form a strong classifier, which effectively enhances classification accuracy and mitigates overfitting. To further improve the stability and accuracy of the model, a voting mechanism was designed to aggregate the prediction results from different time segments. This mechanism applied weighted voting based on the classification results of each time segment, ultimately determining whether a subject was diagnosed with ASD. In this manner, independent information from each time segment was effectively utilized while minimizing the impact of errors from a single time segment on the final decision.

#### 2.3.2. Spatial Feature Extraction

After preprocessing the data using Nilearn, the dimension of the data is $X\in R^{V\times T}$, where *V* represents the number of spatial voxels (with preprocessed brain data having dimensions of 99×117×95), and *t* corresponds to the number of time points in the fMRI data. In the spatial feature extraction process, for each time point’s data $X_{i}\in R^{V}$, we use a 3D Convolutional Neural Network (3D-CNN) to extract the spatial features.

The 3D-CNN extracts spatial features by performing convolution operations on the input data. A three-dimensional convolutional kernel $K\in R^{D\times H\times W}$ is used, where *D*, *H*, and *W* represent the depth, height, and width of the kernel, respectively. During the convolution process, the kernel slides over the 3D image data, performing convolution to capture spatial patterns of brain activity. The spatial information from each time point is processed through 3D convolution to produce the convolved feature map $Y_{i}$. The convolution operation can be expressed as follows:

$$Y_{i}=X_{i}*K$$

where ∗ denotes the convolution operation, *K* is the convolution kernel, and $X_{i}$ is the input data. The output feature map is denoted as $F_{i}$. After each convolutional layer, a pooling operation is applied to reduce the spatial dimensions while preserving important spatial information. The pooling operation can be expressed as follows:

$$Y_{\mathrm{pool}}=\mathrm{MaxPool}\left(Y_{i}\right)$$

To input the feature map into the fully connected layer, we flatten the pooled feature map, transforming the multi-dimensional spatial features into a one-dimensional feature vector:

$$Y_{\mathrm{flat}}=\mathrm{Flatten}\left(Y_{\mathrm{pool}}\right)$$

The flattened feature vector is then passed to the fully connected layer (FC), where a linear transformation maps the features to the target dimension, resulting in the final spatial feature representation $F_{\mathrm{sp},i}$:

$$F_{\mathrm{sp},i}=\mathrm{FC}\left(Y_{\mathrm{flat}}\right)$$

Through this process, after being processed by the 3D Convolutional Neural Network, the spatial features of each time point are extracted as a one-dimensional spatial feature vector. For the input data consisting of *t* time points, we obtain *t* spatial feature representations, $F_{\mathrm{sp},i}\in R^{d_{1}}$, where $d_{1}$ is the dimension of the output spatial features. Finally, the collection of spatial feature vectors for all time points is denoted as $F_{\mathrm{sp}}=\left\{F_{\mathrm{sp},1},F_{\mathrm{sp},2},\ldots ,F_{\mathrm{sp},t}\right\}$.

By using 3D-CNNs, we can extract spatial features from the data of each time point. These features are obtained through convolution, pooling, and flattening operations, providing rich spatial information for further temporal feature extraction and classification. The multi-layer structure of the 3D-CNN enables the network to effectively learn spatial patterns at various levels, thus offering strong support for the analysis of brain functional states.

#### 2.3.3. Temporal Feature Extraction

In the temporal feature extraction module, we use the LSTM model to extract temporal features from the preprocessed data $X\in R^{V\times t}$, capturing the temporal dynamics of brain activity. Specifically, we divide the input data into multiple sliding time windows, where each time window has a length of *w* and a sliding step size of *s*, resulting in *k* time windows. Each time window contains *w* time points, denoted as $X_{j}\in R^{V\times w}$, where *j* represents the index of the time window, *V* is the number of spatial voxels, and *w* is the number of time points in the time window. The number of time windows *k* can be computed using the following formula:

$$k=\left\lfloor \frac{t-w}{s}\right\rfloor +1$$

The choice of a sliding window size (w = 10) and a time window size (t = 50) is to effectively capture the temporal dynamics of brain activity in individuals with Autism Spectrum Disorder (ASD). A window length of 10 times points allows for the capture of short-term neural activity changes, while the overlap of a time point ensures continuity of information and prevents data loss. A time window size of 50 encompasses a sufficiently long duration, aiding in the identification of the complex temporal dependencies required for recognizing ASD characteristics. This configuration enhances the model’s accuracy in identifying patterns of brain activity associated with ASD.

For each time window $X_{j}$, we input it into the LSTM model to extract temporal features. LSTM, a special type of recurrent neural network (RNN), effectively solves the problems of gradient vanishing and exploding that traditional RNNs face when processing long time sequences. LSTM introduces memory cells and gating mechanisms to selectively remember, forget, and update information at each time step, enabling the network to capture long-term dependencies. This is especially important when processing high-dimensional, long time series data like the ABIDE dataset, where LSTM effectively models the temporal dependencies present in the data. The core mechanism of LSTM includes three main gates: the Forget Gate, the Input Gate, and the Output Gate, along with a memory cell. At each time step of the LSTM network, we input one time point data from $X_{j}$, and the hidden state of the LSTM is updated sequentially, ultimately resulting in the temporal feature $F_{\mathrm{tem},j}$ for each time window:

$$F_{\mathrm{tem},j}=\mathrm{LSTM}\left(X_{j}\right)$$

where $F_{\mathrm{tem},j}\in R^{d_{2}}$ is the temporal feature vector output by LSTM, representing the temporal dynamic features of brain activity within that time window, and $d_{2}$ is the dimension of the output temporal features. The LSTM’s gating mechanism efficiently models both short- and long-term dependencies in the time-series data. The dynamic features between different time windows in the ABIDE dataset can reveal potential differences in brain function.

After processing all the time windows with the LSTM, we obtain the set of temporal feature vectors $F_{\mathrm{tem}}=\left\{F_{\mathrm{tem},1},F_{\mathrm{tem},2},\ldots ,F_{\mathrm{tem},k}\right\}$, which will be used in conjunction with the spatial features for the subsequent classification task. In this way, through the LSTM processing, we can extract the temporal features from each time window, capture the temporal dynamics of brain activity, and further enhance the model’s classification ability.

#### 2.3.4. Classification and Decision-Making Module

In the classification decision module, the goal is to determine whether an individual belongs to the ASD group based on the extracted spatiotemporal features. The fMRI data of each subject are divided into *k* time windows, where *k* is chosen to be odd to avoid ties during the voting phase. For each time window, temporal features are extracted using an LSTM model, as described in the temporal feature extraction module. Additionally, for each time window, the temporal features of the current window are concatenated with the spatial features of the first time point within the same window, forming a combined feature vector. These concatenated feature vectors are then input into the GBDT model for classification. Finally, the individual’s ASD status is determined by performing a voting procedure based on the *k* classification results.

**(1)**  **Spatiotemporal Feature Fusion**

For each subject’s preprocessed fMRI data, the data are divided into *k* time windows. For each time window, temporal features $F_{\mathrm{tem},j}\in R^{d_{2}}$ are extracted using the LSTM model, where *j* represents the index of the time window, and $d_{2}$ is the dimensionality of the temporal features. Simultaneously, the spatial features of the first time point in the time window, $F_{\mathrm{sp},1}\in R^{d_{1}}$, are obtained via the 3D-CNN.

**Figure 2:**
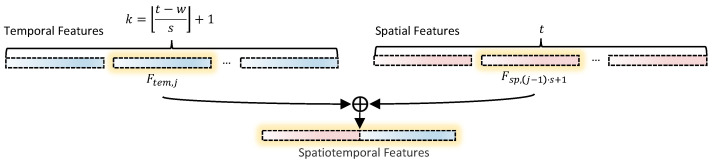
Spatiotemporal feature fusion diagram.

Next, as shown in Figure 2, the temporal feature $F_{\mathrm{tem},j}$ is concatenated with the spatial feature $F_{\mathrm{sp},1}$ from the first time point of the same time window to form a combined feature vector $F_{\mathrm{comb},j}$, with dimensionality $d_{1}+d_{2}$:

$$F_{\mathrm{comb},j}=\mathrm{concat}\left(F_{\mathrm{tem},j},F_{\mathrm{sp},(j-1)\cdot s+1}\right)\in R^{d_{1}+d_{2}}$$

where concat denotes the concatenation operation. The concatenated feature vector $F_{\mathrm{comb},j}$ is then passed as input to the GBDT classifier for classification.

**(2)**  **GBDT Classification**

The GBDT model is used to classify the combined feature vector $F_{\mathrm{comb},j}$ for each time window, determining whether the individual belongs to the ASD group or the control group. The GBDT is an ensemble learning method that iteratively trains decision trees and gradually corrects the errors of previous models. The core advantage of the GBDT model lies in its ability to effectively capture complex nonlinear relationships between features and target variables. By integrating multiple weak classifiers (decision trees), the GBDT not only improves accuracy but also reduces the risk of overfitting. Additionally, the GBDT can naturally handle high-dimensional, sparse features and is robust to correlations between features. As a result, the GBDT is widely used in tasks requiring high accuracy and the ability to handle complex data patterns. Specifically, the GBDT trains decision tree models by optimizing a loss function (typically minimizing mean squared error or log-likelihood loss). For each time window *j*, the output of the GBDT is the predicted classification result for that time window:

$$\hat{y_{j}}=\mathrm{GBDT}\left(F_{\mathrm{comb},j}\right)$$

where ${\hat{y}}_{j}\in \left\{0,1\right\}$ represents the predicted classification result for the *j*th time window.

**(3)**  **Voting Decision**

Once the classification results ${\hat{y}}_{j}$ for the *k* time windows are obtained, we use a majority voting rule to make the final classification decision. The core purpose of the voting decision mechanism is to integrate the temporal information implicit in different time windows, thereby improving the model’s stability and accuracy. Since fMRI signals inherently exhibit high temporal characteristics, the data within each time window represent a phase of brain activity, and different time windows may reflect the brain’s functional state at different time points. Through the voting decision, we can better integrate these phase-based information and avoid the bias that may arise from the classification result of a single time window. Specifically, the majority voting rule determines the final classification of the subject based on the classification results of the *k* time windows. The final majority voting result ${\hat{y}}_{\mathrm{final}}$ is computed as follows:

$${\hat{y}}_{\mathrm{final}}=\mathrm{vote}\left(\hat{y_{1}},\hat{y_{2}},\ldots ,\hat{y_{k}}\right)$$

where vote represents the voting function that returns the class label that appears more than half the time. Specifically, the final voting result is calculated using the following formula:

$${\hat{y}}_{\mathrm{final}}=\left\{\begin{array}{cc} 1 & \mathrm{if}\;\sum_{j=1}^{k}{\hat{y}}_{j}>\frac{k}{2}\\ 0 & \mathrm{if}\;\sum_{j=1}^{k}{\hat{y}}_{j}\leq \frac{k}{2} \end{array}\right.$$

In this way, the final classification result ${\hat{y}}_{\mathrm{final}}$ is determined by the majority of the classification results from the *k* time windows. Since *k* is an odd number, we ensure that no ties occur in the majority voting decision.

To train the GBDT model, we use the combined feature vector $F_{\mathrm{comb},j}$ for each time window as input and the corresponding ground truth label $y_{j}\in \left\{0,1\right\}$ as the target output. The GBDT model is trained by optimizing the cross-entropy loss function, using gradient descent to minimize the prediction error on the training dataset. Let $D={\left\{\left(F_{\mathrm{comb},j},y_{j}\right)\right\}}_{j=1}^{k}$ be the training dataset, where each feature vector $F_{\mathrm{comb},j}$ is paired with the true label $y_{j}$. The loss function used is binary cross-entropy:

$$L=-\sum_{j=1}^{k}\left[y_{j}\log\left(p_{j}\right)+\left(1-y_{j}\right)\log\left(1-p_{j}\right)\right]$$

where $p_{j}$ is the classification probability for the *j*th time window. After training, the GBDT model can be used to predict new subjects’ data. During prediction, the same feature extraction process as training is used to compute the combined feature vector, which is then input into the trained GBDT model for classification. The final classification decision is obtained by majority voting of the classification results from the *k* time windows, determining whether the individual belongs to the ASD group or the control group. A classification result of 1 indicates an individual with ASD, while 0 indicates a typical control.

This section presents the detailed methodology of the proposed approach for the identification of ASD. First, the fMRI data are preprocessed using Nilearn to ensure high-quality preprocessing, maintaining both a signal-to-noise ratio and spatial consistency in the input data. Next, a 3D-CNN is employed to extract spatial features from the data at each time point, effectively capturing functional connectivity patterns across different brain regions. Subsequently, an LSTM model is used to process the temporal data, allowing for the exploration of the dynamic properties of brain activity. Finally, we design a classification decision module based on the fusion of spatial and temporal features, where GBDTs are used to classify the integrated spatiotemporal features. A voting mechanism is introduced to enhance the stability and accuracy of the classification results.

The combination of spatiotemporal feature fusion and the voting mechanism provides significant advantages for ASD recognition. First, spatiotemporal feature fusion fully leverages both spatial and temporal information, thereby enhancing the model’s discriminative power. The 3D-CNN-based spatial feature extraction captures activity patterns across different brain regions, while the LSTM-based temporal feature extraction focuses on the dynamic changes in the time series, with both methods complementing each other to more comprehensively represent ASD characteristics. Additionally, the voting mechanism integrates predictions from different time segments with weighted averaging, effectively reducing errors that might arise from individual time windows, further improving the robustness and accuracy of the model. This innovative spatiotemporal information fusion approach holds significant potential for brain imaging analysis in ASD and provides an effective and reliable solution for early ASD identification.

## 3. Results

To evaluate the performance of the proposed model, this section presents the experimental results on the datasets from 17 different sites in the ABIDE I dataset. The key performance metrics of the proposed method, including accuracy, recall, precision, F1-score, and specificity, are reported. In addition, this section compares the performance of different methods on the same datasets and provides a further analysis of the strengths and weaknesses of each approach. To comprehensively assess the classification performance of the proposed model, several commonly used evaluation metrics were employed, including accuracy, precision, recall, F1-score, and specificity. These metrics allow us to measure the model’s performance from different perspectives.

### 3.1. Performance Metrics

**(1)**  **Accuracy**

Accuracy represents the proportion of correctly predicted samples by the classification model. It is calculated as follows:

$$\mathrm{Accuracy}=\frac{TP+TN}{TP+TN+FP+FN}$$

where TP (True Positive) is the number of samples that are truly positive and predicted as positive, TN (True Negative) is the number of samples that are truly negative and predicted as negative, FP (False Positive) is the number of samples that are truly negative but predicted as positive, and FN (False Negative) is the number of samples that are truly positive but predicted as negative.

**(2)**  **Precision**

Precision measures the proportion of predicted positive samples that are actually positive, reflecting the accuracy of the model in predicting the positive class. It is calculated as follows:

$$\mathrm{Precision}=\frac{TP}{TP+FP}$$

**(3)**  **Recall**

Recall, also known as Sensitivity, measures the proportion of actual positive samples that the model correctly identifies as positive, reflecting the model’s ability to capture positive class samples. It is calculated as follows:

$$\mathrm{Recall}=\frac{TP}{TP+FN}$$

**(4)**  **F1-score**

The F1-score is the harmonic mean of precision and recall, providing a comprehensive evaluation of the model’s precision and recall capabilities. The F1-score is particularly useful in cases of class imbalance as it balances precision and recall. It is calculated as follows:

$$F1\text{-}\mathrm{score}=2\times \frac{\mathrm{Precision}\times \mathrm{Recall}}{\mathrm{Precision}+\mathrm{Recall}}$$

**(5)**  **Specificity**

Specificity measures the proportion of actual negative samples that the model correctly identifies as negative, reflecting the model’s ability to identify negative class samples. It is calculated as follows:

$$\mathrm{Specificity}=\frac{TN}{TN+FP}$$

These evaluation metrics provide a comprehensive reflection of the model’s classification performance. In particular, in the task of Autism Spectrum Disorder (ASD) recognition, metrics such as accuracy, recall, and F1-score offer important reference points for model evaluation.

### 3.2. Experimental Setup

In this study, all experiments were conducted using the PyTorch 1.11.0 framework and ran on a high-performance Nvidia 3090 GPU to ensure efficient model training and evaluation. To comprehensively assess the performance of the proposed model, a *k*-fold cross-validation strategy was employed. Specifically, in the single-site classification task, the dataset was randomly divided into *k* equally sized subsets. In each experiment, one subset was selected as the test set, while the remaining four subsets were used as the training set. This approach ensured that each subset was used as the test set in one of the *k* experiments, minimizing data partition bias and enhancing the generalizability of the experimental results. The procedure was repeated *k* times, with a different subset used as the test set each time. Finally, the evaluation metrics from the *k* experiments were averaged to yield the model’s final performance results.

For spatial feature extraction, a 3D CNN model with two convolutional layers was employed. In each layer, multiple parallel convolutional kernels of sizes 3×3×3, 5×5×5, 7×7×7 were applied to capture multi-scale spatial patterns. The outputs of these parallel convolutions were aggregated to form a unified feature representation. Following each convolutional layer, a 3D max-pooling layer with a kernel size of 2 and a stride of 2 was used to progressively reduce the spatial dimensions while preserving salient features. For temporal feature extraction, an LSTM model was used with 500 hidden units. The learning rate was set to 0.001, and the batch size was set to 32. The model was trained for 50 epochs.

In this experiment, the dataset’s time window size was set to t=50. For hyperparameter tuning, the sliding window size was set to w=10, and the sliding step was set to s=1.

### 3.3. Experimental Results

In this section, we present the experimental results of multiple configurations to validate the effectiveness and superiority of the proposed methods.

#### 3.3.1. Performance Comparison of Different Model Configurations

In this study, to comprehensively evaluate the performance of the proposed **3D-CNN + LSTM + GBDT** model and compare it with other commonly used combinations, we designed several experiments. Specifically, we compared the following model configurations: (1) **3D-CNN + LSTM + GBDT**, which is our proposed method; (2) **3D-CNN + GBDT**; (3) **3D-CNN + RNN + GBDT**; (4) **3D-CNN + LSTM + Random Forest (RF)**; (5) **3D-CNN + RNN + RF**. These models were evaluated using five-fold and ten-fold cross-validation strategies for accuracy. By comparing these configurations, we aim to validate the advantages of the selected model. Configuration (2) was used to verify the effectiveness of temporal–spatial feature fusion, while configurations (3), (4), and (5) aimed to determine the optimal choice for temporal feature extraction and classification strategies.

**Figure 3:**
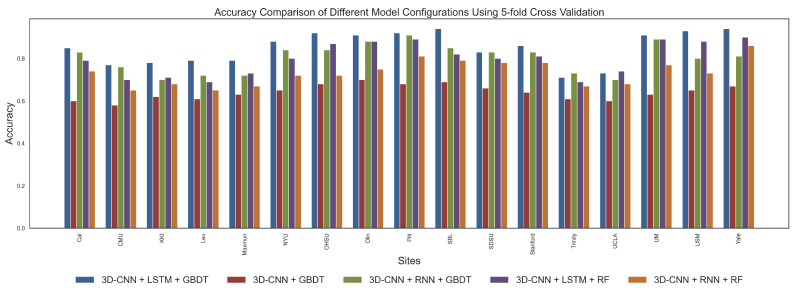
Accuracy comparison of different models across 17 sites under 5-fold cross-validation.

**Figure 4:**
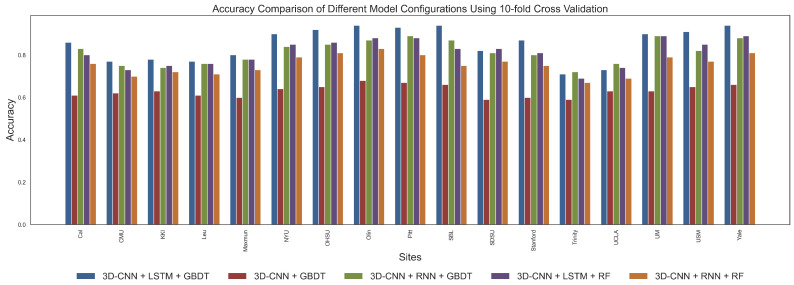
Accuracy comparison of different models across 17 sites under 10-fold cross-validation.

Through five-fold and ten-fold cross-validation, we obtained the average classification results for different models. Figure 3 and Figure 4 present the classification accuracy for each site under different model configurations, while Table 3 and Table 4 show the average values of precision, recall, accuracy, F1-score, and specificity for each site under the various model configurations. As shown in figures and tables, **3D-CNN + LSTM + GBDT** consistently achieved the best average classification performance across all model configurations. Specifically, this model achieved an accuracy of 0.85 in both five-fold and ten-fold cross-validation, with only minimal differences observed between the two methods. Additionally, **3D-CNN + LSTM + GBDT** significantly outperformed **3D-CNN + GBDT** in terms of accuracy, recall, and F1-score. This indicates that the **3D-CNN + LSTM + GBDT** model is highly effective in capturing temporal information, and the fusion of spatial and temporal features further enhances classification accuracy. More specifically, temporal–spatial feature fusion allows the model to extract spatial features at each time point and capture dynamic brain activity changes, thus providing a more comprehensive feature representation and improving classification performance. In contrast, the models using other combinations of temporal models and classifiers had lower accuracy, particularly when using RNN and RF, with performance notably decreasing. This suggests that the combination of LSTM and GBDT has a unique advantage in capturing temporal dependencies and nonlinear relationships. The results from both five-fold and ten-fold cross-validation demonstrate that although the validation methods differ slightly, the relative ranking of the configurations remains consistent, with **3D-CNN + LSTM + GBDT** consistently outperforming all other configurations in terms of stability and classification efficiency.

The performance differences between the selected model configurations are substantial. First, **3D-CNN + LSTM + GBDT** significantly outperforms all other combinations across all metrics. This combined model integrates the spatial feature extraction capability of 3D-CNN, the temporal dependency modeling advantage of LSTM, and the efficient nonlinear classification power of GBDT. Specifically, 3D-CNN extracts spatial information from brain images through convolution operations, LSTM captures long-term dependencies in the time series, and GBDT optimizes classification performance by integrating decision trees. Compared to other combinations, LSTM is better at handling the complexity of temporal features in ASD data, and GBDT is more efficient in handling nonlinear relationships between temporal and spatial features, making **3D-CNN + LSTM + GBDT** the optimal choice.

In contrast, **3D-CNN + RNN + GBDT** performed slightly worse. Although RNNs can handle sequential data, they have limitations in capturing long-term dependencies, especially when dealing with longer time series, where issues such as vanishing or exploding gradients may occur. Therefore, the inability of RNN to effectively model long-term dependencies results in lower classification performance compared to LSTM. On the other hand, the **3D-CNN + LSTM + RF** model, while performing well on some metrics, is less effective than GBDT. RF, as an ensemble method based on decision trees, is typically not as efficient as GBDT in handling high-dimensional data. GBDT optimizes decision boundaries better, leading to superior performance compared to RF. Hence, **3D-CNN + LSTM + GBDT** is better suited to handle complex classification tasks. Finally, **3D-CNN + RNN + RF** performed the worst, especially in terms of accuracy and recall, which were significantly lower than other models. The combination of RNN and RF did not effectively leverage the strengths of both methods, leading to poor performance in processing the ABIDE dataset.

Through a series of experiments and comparisons, we found that the **3D-CNN + LSTM + GBDT** model performed best across all configurations, especially in key metrics such as accuracy, precision, and recall. This model effectively combines the spatial feature extraction capability of 3D-CNN, the temporal dependency modeling of LSTM, and the nonlinear classification power of GBDT, making it highly effective in processing the complex temporal-spatial features in the ABIDE dataset. For ASD classification tasks, the combination of LSTM and GBDT proves to be the optimal choice, further validating the effectiveness of supervised learning methods in processing brain imaging data.

#### 3.3.2. Comparison with Other Studies

In this section, we compare our proposed method with other state-of-the-art approaches to evaluate its performance in the single-site classification task. Table 5 presents the classification accuracy results for various algorithms, including **Epalle** [14], **Heinsfeld** [11], **Eslami** [12], **Nielsen** [10], **Rakić** [13], and **Kang** [15]. The table lists the classification results for each site, along with the accuracy of each method at individual sites.

From Table 5, it is evident that our proposed method outperforms other methods at multiple sites, with an overall average accuracy of 0.85, which is significantly higher than **Heinsfeld** [11] with 0.65 and **Nielsen** [10] with 0.60, and also surpasses **Rakić** [13] with 0.80 and **Kang** [15] with 0.83. At several sites, including Cal, Leu, NYU, Olin, Pitt, SBL, and UM, our method performs exceptionally well, achieving accuracies of 0.83, 0.83, 0.90, 0.89, 0.93, 0.96, and 0.92, respectively, all surpassing the performance of the other methods. Although the improvement over **Kang** [15]’s method is relatively modest, our approach demonstrates enhanced robustness, with lower variance across folds (±0.03 vs. ±0.06), suggesting better generalization and stability across heterogeneous sites.

The performance improvement of our model can be attributed to the effective combination of 3D-CNN, LSTM, and GBDT. Notably, 3D-CNN is capable of effectively extracting spatial features from brain imaging data, capturing voxel-level details of brain activity. LSTM enhances the model’s ability to handle time-series data by modeling temporal dependencies, with a particular advantage in capturing long-term dependencies. Lastly, GBDT leverages decision tree ensembles to handle complex nonlinear relationships, further optimizing classification performance.

While most sites show excellent performance, some sites such as CMU, SDSU, and Trinity exhibit relatively lower performance. For instance, at the CMU site, our method achieves an accuracy of 0.77, which is still higher than **Heinsfeld’s** [11] 0.66 but lower than **Epalle** [14]. This performance variation could be attributed to differences in site data characteristics, such as sample size, fMRI data quality, and noise levels. Nevertheless, despite these discrepancies at individual sites, our method consistently demonstrates strong competitive performance, especially when dealing with challenging datasets, providing reliable classification results. To further explore the lower classification performance observed at certain sites, we examined the relationship between misclassification and individual-level variables such as age and head motion. While no clear age-related trends were found, we observed that misclassified subjects tended to have higher mean framewise displacement, suggesting that motion-related artifacts may have negatively impacted model predictions.

Table 6 presents the comparative performance of different methods for multi-site classification. As indicated in the table, the proposed method exhibits superior performance across several evaluation metrics, including accuracy, precision, recall, F1-score, and specificity. Our method outperforms all other approaches in most of these metrics, showing improvements in accuracy, recall, F1 score, and specificity when compared to the state-of-the-art method. This highlights its robust generalization capability across multi-site datasets. Notably, our method excels in recall, demonstrating high sensitivity in identifying ASD samples.

#### 3.3.3. The T-SNE Visualization of Features

**Figure 5:**
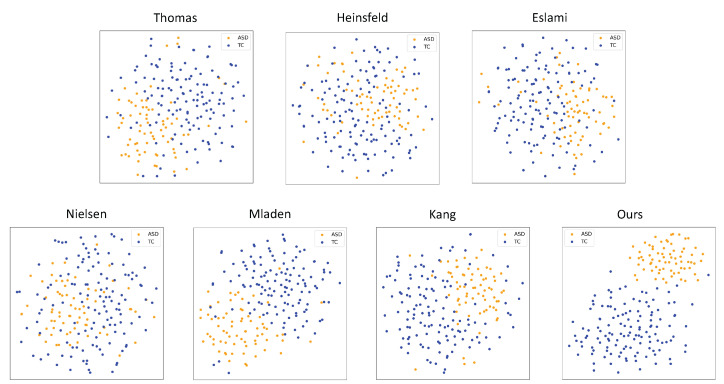
T-SNE visualization of features at the Olin site.

In this experiment, we compare the feature distributions extracted by our proposed method with those of other methods at the Olin site. To facilitate a more intuitive comparison of the classification performance of each method, we employed T-Distributed Stochastic Neighbor Embedding (T-SNE) for dimensionality reduction and visualization of the features. Figure 5 presents the feature distributions of each method after dimensionality reduction.

As shown in Figure 5, the feature distribution of our method clearly demonstrates a better separation between positive and negative samples. Specifically, the overlap between the positive and negative samples is minimal, forming distinct clusters. This indicates that the fusion of spatiotemporal features effectively enhances the model’s ability to distinguish between different classes, especially between ASD and normal control groups, showcasing strong separability.

In contrast, the feature distributions of the other methods are more overlapped. While some methods exhibit partial separation between the positive and negative samples, the overall degree of separation is not as clear as in our method. For instance, in the methods by **Heinsfeld’s** [11], **Eslami** [12], and **Nielsen** [10], the positive and negative samples overlap significantly, making it challenging to achieve good classification performance. Other methods, such as **Epalle** [14], **Rakić** [13], and **Kang** [15], show some improvement in separation but still lack sufficient distinction between the positive and negative samples compared to our proposed method.

This phenomenon can be attributed to the advantages of our spatiotemporal feature fusion approach. By using 3D-CNN to extract spatial features and combining it with LSTM for modeling temporal information, we can more comprehensively capture the dynamic changes in brain activity over time, rather than solely relying on static spatial features or local temporal features. This spatiotemporal fusion allows the model to not only accurately extract spatial information from brain images but also to capture the temporal dynamics of brain activity, thus improving the model’s ability to differentiate between ASD and normal control groups.

### 3.4. Discussion

The experimental results of this study demonstrate that the proposed 3D-CNN with segmental temporal decision network method for ASD recognition exhibits superior performance in ASD classification tasks, particularly in handling spatiotemporal features. The fusion of spatiotemporal features is a key aspect of this method, which significantly enhances the model’s discriminative power in the classification task. By utilizing 3D-CNN for spatial feature extraction, LSTM for modeling temporal dependencies, and combining the nonlinear classification ability of GBDT, the model is able to comprehensively capture the complex patterns of brain activity across both spatial and temporal dimensions, thereby optimizing classification performance.

Firstly, the 3D-CNN effectively extracts spatial structural information from fMRI data, capturing spatial correlations between different brain regions. LSTM, on the other hand, captures the dynamic changes in brain activity over time by modeling long-term dependencies in time-series data. Compared to traditional methods that rely solely on spatial information or simple temporal features, the fusion of spatiotemporal features enables the model to not only extract spatial features more accurately but also handle the temporal variations in brain activity, enhancing the model’s ability to distinguish between ASD and typically developing controls.

Compared to other methods, our spatiotemporal fusion approach significantly improves the separability of the features. In particular, the T-SNE visualization results clearly show a substantial reduction in the overlap between positive and negative samples, indicating that our method effectively distinguishes between different classes in the feature space. This improved feature separability directly contributes to better classification performance across multiple sites, particularly at the Cal, NYU, and Olin sites, where our method achieves higher accuracy than other methods.

In this study, we used T-SNE visualization to demonstrate the separation between the autism spectrum disorder (ASD) group and the control group, but we did not identify the specific features driving this separation. Understanding these features is crucial for a deeper exploration of the biological mechanisms underlying ASD. Therefore, future research will incorporate techniques such as feature importance analysis and SHAP values to identify and discuss the potential associations between key features and ASD. Additionally, we will strive to enhance the transparency of the model to ensure its effectiveness and interpretability in clinical applications, thereby providing insights for understanding the causes of ASD and supporting early intervention.

Furthermore, the voting mechanism employed in this study further enhances the model’s classification performance. The voting mechanism combines the predictions from different models, leveraging the understanding of spatiotemporal features from each model to increase the robustness of the final classification result. Specifically, the introduction of the voting mechanism effectively mitigates errors that may arise from a single model when confronted with challenging samples, thus improving both classification accuracy and stability. In practical applications, this strategy aggregates information across multiple time windows, ensuring that the model’s predictions at each stage reflect the full scope of the data, thereby producing more reliable results.

The recall rate of the proposed model in this method ranges from 0.83 to 0.85, indicating that some ASD cases may have been missed. We recognize that false-negative results can lead to missed early identification of individuals who require intervention, which could impact their long-term development and treatment outcomes. To improve the model’s recall rate, we plan to explore additional features and variables in future research to enhance the detection capabilities for ASD. We will also consider adjusting the model’s threshold settings, employing different algorithms, or integrating multiple methods to bolster the recall rate and minimize the risk associated with false negatives.

In clinical applications, we recommend using our model as an auxiliary tool in conjunction with traditional assessment methods. While the model can support early identification, the final diagnosis should still be made by qualified healthcare professionals to ensure that each individual receives a comprehensive evaluation and the necessary support.

However, despite the outstanding performance of our model across most sites, there are still some sites where the performance is relatively lower. This performance variability could be attributed to differences in data characteristics between sites, such as sample size, data quality, and noise levels. To address these issues, future work could explore further optimization of data preprocessing and noise removal methods to improve the model’s performance at these sites.

Although our model performs excellently in terms of accuracy, its computational complexity may pose challenges for real-time clinical applications, particularly when constrained by computational resources for larger datasets. To enhance clinical usability, future research should consider model optimization strategies to reduce computational demands or shorten training time through parameter adjustments and optimization of algorithms to improve the feasibility of practical applications.

Current clinical ASD diagnosis largely depends on subjective behavioral evaluations, which can be time-consuming and variable. Our method provides an objective neuroimaging-based approach to complement clinical assessments, potentially improving diagnostic efficiency and accuracy. In the context of neuroimaging-based ASD classification, an accuracy threshold of approximately 70–75% is generally regarded as clinically meaningful, given the inherent heterogeneity of ASD and variability in fMRI data quality across sites. Our proposed model consistently achieves classification accuracies above this benchmark across multiple independent datasets, demonstrating robustness and potential clinical value. Overall, the experimental results validate the effectiveness of the proposed method in ASD classification tasks, demonstrating its strong classification capability, particularly in handling complex spatiotemporal features.

## 4. Conclusions

In this study, we proposed a method for ASD recognition based on a 3D-CNN and segmented LSTM, demonstrating excellent performance in the ASD classification task across multiple sites. By combining spatiotemporal feature extraction and a multi-model voting mechanism, our approach effectively captures both the spatial structure information and the temporal dynamics of brain activity. The proposed model achieved an average classification accuracy of 85%, outperforming several state-of-the-art methods and showing strong generalizability across sites, providing a more comprehensive solution for ASD identification.

Our findings show that utilizing the 3D-CNN to extract high-dimensional spatial features, combined with LSTM to capture the long-term dependencies in time-series data, can better reveal the characteristic brain activity patterns in ASD patients. Furthermore, the GBDT classifier enhances the model’s nonlinear classification capability, and the integration of the voting mechanism significantly improves the robustness and accuracy of the model. With this approach, we were able to effectively differentiate ASD patients from typical controls, achieving high classification accuracy, especially at challenging sites.

Although our study has made considerable progress, several challenges remain. Firstly, the performance variation across sites may be attributed to data heterogeneity, sample size differences, and variations in preprocessing protocols. Future work will focus on optimizing and standardizing preprocessing strategies as well as enhancing model generalization to improve stability and applicability across diverse datasets. Secondly, while the proposed spatiotemporal feature extraction and voting mechanisms have demonstrated efficacy in ASD classification, the high dimensionality and abstract nature of the extracted features pose challenges for biological interpretability. Developing methods to map these learned features onto meaningful neurobiological constructs will be an important direction for future research. Lastly, the framework introduced here provides promising insights for the identification of other neurodevelopmental disorders, such as ADHD or schizophrenia, and could be adapted accordingly to broaden its clinical utility.

Overall, the results of this study provide a new approach for the automated diagnosis of ASD, particularly in the modeling of spatiotemporal features. In the future, we will continue to explore advanced deep learning techniques, incorporate larger and more diverse datasets, and further improve the accuracy and generalizability of the model.

## Figures and Tables

**Table 1:** Class membership information of ABIDE-I dataset for each individual site (part 1).

Site	Caltech	CMU	KKI	Leuven	MaxMun	NYU	OHSU	OLIN	PITT
ASD	19	14	20	29	24	75	12	19	29
Healthy control	18	13	28	34	28	100	14	15	27
Male count	29	21	36	55	48	139	26	29	48
Female count	8	6	12	8	4	36	0	5	8
Average age	27	26	10	18	25	15	10	16	18

**Table 2:** Class membership information of ABIDE-I dataset for each individual site (part 2).

Site	SBL	SDSU	Stanford	Trinity	UCLA	UM	USM	Yale
ASD	15	14	19	22	54	66	46	28
Healthy control	15	22	20	25	44	74	25	28
Male count	30	29	31	47	86	113	71	40
Female count	0	7	8	0	12	27	0	16
Average age	34	14	9	16	13	14	22	12

**Table 3:** Comparison of the average performance of different models across 17 sites under 5-fold cross-validation with confidence intervals.

Configuration	Accuracy (Mean ± CI)	Precision (Mean ± CI)	Recall (Mean ± CI)	F1-Score (Mean ± CI)	Specificity (Mean ± CI)
**3D-CNN + LSTM + GBDT**	0.85 ± 0.03	0.88 ± 0.02	0.83 ± 0.02	0.85 ± 0.01	0.87 ± 0.02
**3D-CNN + GBDT**	0.64 ± 0.04	0.66 ± 0.05	0.59 ± 0.03	0.68 ± 0.04	0.63 ± 0.02
**3D-CNN + RNN + GBDT**	0.80 ± 0.02	0.82 ± 0.03	0.75 ± 0.01	0.78 ± 0.03	0.82 ± 0.04
**3D-CNN + LSTM + RF**	0.80 ± 0.02	0.83 ± 0.03	0.77 ± 0.02	0.80 ± 0.02	0.84 ± 0.06
**3D-CNN + RNN + RF**	0.77 ± 0.03	0.79 ± 0.02	0.72 ± 0.02	0.75 ± 0.03	0.80 ± 0.05

**Table 4:** Comparison of the average performance of different models across 17 sites under 10-fold cross-validation with confidence intervals.

Configuration	Accuracy (Mean ± CI)	Precision (Mean ± CI)	Recall (Mean ± CI)	F1-Score (Mean ± CI)	Specificity (Mean ± CI)
**3D-CNN + LSTM + GBDT**	0.85 ± 0.03	0.87 ± 0.04	0.85 ± 0.03	0.86 ± 0.05	0.89 ± 0.03
**3D-CNN + GBDT**	0.63 ± 0.04	0.65 ± 0.03	0.60 ± 0.05	0.62 ± 0.04	0.72 ± 0.06
**3D-CNN + RNN + GBDT**	0.81 ± 0.03	0.81 ± 0.04	0.82 ± 0.03	0.81 ± 0.05	0.85 ± 0.04
**3D-CNN + LSTM + RF**	0.81 ± 0.04	0.83 ± 0.05	0.81 ± 0.05	0.82 ± 0.06	0.87 ± 0.05
**3D-CNN + RNN + RF**	0.77 ± 0.03	0.78 ± 0.06	0.77 ± 0.03	0.77 ± 0.05	0.83 ± 0.04

**Table 5:** Single-site classification results for different methods under 5-fold cross-validation.

Site	Epalle	Heinsfeld	Eslami	Nielsen	Rakić	Kang	Ours
**Cal**	0.74	0.68	0.53	0.49	0.74	0.82	0.83
**CMU**	0.81	0.66	0.69	-	0.60	-	0.77
**KKI**	0.78	0.67	0.70	0.55	0.76	-	0.79
**Leu**	0.75	0.65	0.61	0.61	0.79	0.61	0.83
**Maxmun**	0.72	0.68	0.49	-	0.82	-	0.79
**NYU**	0.81	0.66	0.68	0.60	0.87	0.84	0.90
**OHSU**	0.79	0.64	0.82	0.43	0.74	0.93	0.92
**Olin**	0.82	0.64	0.65	0.54	0.86	0.80	0.89
**Pitt**	0.74	0.66	0.68	0.65	0.85	0.92	0.93
**SBL**	0.80	0.66	0.52	0.69	0.67	0.92	0.96
**SDSU**	0.68	0.63	0.63	0.65	0.80	-	0.83
**Stanford**	0.69	0.66	0.64	0.68	0.81	-	0.86
**Trinity**	0.68	0.65	0.54	0.46	0.82	0.69	0.77
**UCLA**	0.74	0.66	0.73	0.56	0.90	0.76	0.74
**UM**	0.83	0.64	0.64	0.52	0.87	0.90	0.92
**USM**	0.68	0.64	0.68	0.69	0.87	0.98	0.92
**Yale**	0.77	0.64	0.64	0.52	0.93	0.80	0.86
**Average**	0.77	0.65	0.64	0.60	0.80	0.83	0.85

**Table 6:** Multi-site classification results for different methods under 5-fold cross-validation.

Methods	Accuracy	Precision	Recall	F1-Score	Specificity	*p*-Value
**Thomas**	0.78	0.76	0.78	0.77	0.77	0.015
**Heinsfeld**	0.70	0.67	0.74	0.70	0.63	<0.001
**Eslami**	0.70	0.69	0.69	0.69	0.72	<0.001
**Nielsen**	0.56	0.56	0.57	0.56	0.54	<0.001
**Mladen**	0.79	0.74	0.77	0.75	0.78	0.010
**Kang**	0.83	0.80	0.82	0.81	0.81	0.045
**Ours**	0.84	0.76	0.88	0.82	0.82	-

## Data Availability

The data used in this study were obtained from the publicly available ABIDE I dataset, which can be accessed at https://fcon_1000.projects.nitrc.org/indi/abide/abide_I.html (accessed on 10 November 2024).
